# In Situ Preparation of Three-Dimensional Porous Nickel Sulfide as a Battery-Type Supercapacitor

**DOI:** 10.3390/molecules28114307

**Published:** 2023-05-24

**Authors:** Qixun Xia, Lijun Si, Keke Liu, Aiguo Zhou, Chen Su, Nanasaheb M. Shinde, Guangxin Fan, Jun Dou

**Affiliations:** 1 School of Materials Science and Engineering, Henan Polytechnic University, Jiaozuo 454003, China212006010012@home.hpu.edu.cn (L.S.);; 2 Department of Chemical Engineering (BK21 FOUR), Dong-A University, 37 Nakdong-daero, Saha-gu, Busan 49315, Republic of Korea; 3 Postdoctoral Workstation in LB Group Co., Ltd., Jiaozuo 454000, China

**Keywords:** nickel sulfide, supercapacitor, crystal growth, porous materials

## Abstract

A one-step sulfurization method to fabricate Ni_3_S_2_ nanowires (Ni_3_S_2_ NWs) directly on a Ni foam (NF) was developed as a simple, low-cost synthesis method for use as a supercapacitor (SC), aimed at optimizing energy storage. Ni_3_S_2_ NWs have high specific capacity and are considered a promising electrode material for SCs; however, their poor electrical conductivity and low chemical stability limit their applications. In this study, highly hierarchical three-dimensional porous Ni_3_S_2_ NWs were grown directly on NF by a hydrothermal method. The feasibility of the use of Ni_3_S_2_/NF as a binder-free electrode for achieving high-performance SCs was examined. Ni_3_S_2_/NF exhibited a high specific capacity (255.3 mAh g^−1^ at a current density of 3 A g^−1^), good rate capability (2.9 times higher than that of the NiO/NF electrode), and competitive cycling performance (capacity retention of specific capacity of 72.17% after 5000 cycles at current density of 20 A g^−1^). Owing to its simple synthesis process and excellent performance as an electrode material for SCs, the developed multipurpose Ni_3_S_2_ NWs electrode is expected to be a promising electrode for SC applications. Furthermore, the synthesis method of self-growing Ni_3_S_2_ NW electrodes on 3D NF via hydrothermal reactions could potentially be applied to the fabrication of SC electrodes using a variety of other transition metal compounds.

## 1. Introduction

Supercapacitors (SCs) have garnered considerable attention in the field of next-generation electronics, which include various portable electronic devices, autonomous electric vehicles, and roll-up displays. This may be attributed to their excellent performance in terms of power density, charge/discharge rates and cycling stability. SCs possess higher energy density, a faster charging/discharging rate (within a few seconds), much greater power density, and a longer cycle life than rechargeable batteries [1,2,3,4,5]. Transitional metal oxides, such as RuO_2_ [6], NiO [7], MnO_2_ [8], and their mixed oxides, have emerged as potential candidates for SC electrode materials [9,10,11,12]. This can be attributed to their small band gap and abundant active sites, which result in better electronic conductivity and higher theoretical capacity than that of mono-metal oxides [13]. Among the transition metal oxides, nickel-based oxides are widely used to prepare SC electrodes, owing to their high theoretical specific capacitance [14,15,16,17,18], abundance, and easy preparation methods. Further, compared to metal oxides, metal sulfides exhibit superior electrical conductivity and higher electrochemical activity. Although certain metal sulfides do not have a band gap or electronic structure, they exhibit typical metallic behaviors, which in turn lead to fast redox reaction kinetics [19]. However, the low electron conductivity and non-robust nanostructure of NiO (80.09% capacitance retention after 10,000 cycles) lead to poor rate capability and cycling stability [20]. The electrical conductivity of NiS is at least two orders of magnitude higher than that of NiO and, hence, the former provides outstanding electrochemical activity [20]. Moreover, the performance of electrochemical devices is known to be drastically influenced by their architectures. Among micro/nanomaterials for SCs, materials with one-dimensional (1D) or two-dimensional (2D) architectures with high aspect ratios, low densities, and high surface areas have garnered considerable attention. Cai et al. [21] reported a honeycomb-like nickel–manganese sulfide (NMS) composite nanosheet with a high specific capacity of 205 mAh g^−1^ (at 2 mA cm^−2^). Wei et al. [22] synthesized α-NiS hollow-sphere electrodes with a specific capacitance of 562.3 F g^−1^ (at 0.6 A g^−1^). Wang et al. [23] developed a two-step strategy to synthesize an Ni_x_S_y_ (Ni_3_S_4_ and NiS_2_)/MoS_2_ hybrid nanomaterial for use as a high-performance electrode. The loose structure of this hybrid nanomaterial favors ion diffusion. When utilized as an electrode, the as-synthesized electrode exhibited a high specific capacity (169.44 mAh g^−1^ at 1 A g^−1^), excellent cycling stability (≈88.24% capacity retention after 8000 charge/discharge tests), and excellent rate performance (rate performance of 64.9% for 1–20 A g^−1^). Wang et al. [24] synthesized rigid three-dimensional (3D) Ni_3_S_4_ nanosheet frames assembled from ultrathin NW via a facile solvothermal method. Compared to flat Ni_3_S_4_ sheets, the 3D Ni_3_S_4_ nanosheet frames possess a larger free volume and higher compressive strength, in addition to being able to deliver an exceptional specific capacitance of 1213 F g^−1^. The electrochemical storage principle of energy storage materials—closely related to the types of ions present in the electrolytes. Mu et al. [25] combined density functional theory (DFT) calculations and an in situ X-ray diffraction (XRD) technique to investigate the charge-storage mechanism of MXene in H_2_SO_4_ and confirmed that the c-lattice of Ti_3_C_2_ changes with H^+^ intercalation because of the steric effect in conjunction with the action of the electrostatic force. By combining first-principle calculations and the implicit solvation model and investigating the electronic states during the charge process, Ando et al. [26] proposed the interaction between partially intercalated dehydrated cations and MXene layers. Chen et al. [27] deduced that the charge–storage mechanism of V_2_C may be closely related to the size of cations; cations with smaller radii, such as Mg^2+^ and Li^+^, were intercalated into the interlayer space, and they interacted with the surface V atoms and termination groups. In contrast, larger ions such as Na^+^ and K^+^ could only access a few shallow sites, thereby contributing to superior rate and cycling performance. Hence, 1 M KOH solution was selected as the electrolyte in this work.

However, challenges still remain in the practical application of nickel sulfide; novel structures are necessary to improve the energy storage efficiency [28]. Metal sulfides commonly exhibit superior electrical conductivity and higher electrochemical activity compared to metal oxides. Among metal sulfides, nickel-based sulfides are regarded as particularly attractive because of their enhanced conductivity, abundant redox sites, high structural robustness, and mechanical strength.

In order to achieve the aforementioned objective, we fabricated Ni_3_S_2_ NW supported on Ni foam (NF) via a one-step sulfurization process. The Ni_3_S_2_ NW was directly formed on the conducting NF, which possesses a large active surface area responsible for rapid electrolyte–ion transportation. Our experimental results demonstrated that Ni_3_S_2_ NW exhibits excellent conductivity and a yielding structure, which play key roles in high-performance electrode materials. To develop high-performance asymmetric SC (ASC) devices, we deposited and fabricated Ni_3_S_2_ NW and MXene nanostructures that can be used as electrodes without any conductive additives or binders. Notably, the 3D NF substrates provide a unique structure for the SC electrode, which makes it suitable for applications in energy devices. This advantage motivated us to fabricate a 3D Ni_3_S_2_/NF electrode with a well-arranged nanostructure. The developed Ni_3_S_2_ NW can serve as a SC electrode with excellent stability.

## 2. Results and Discussion

**Figure 1:**
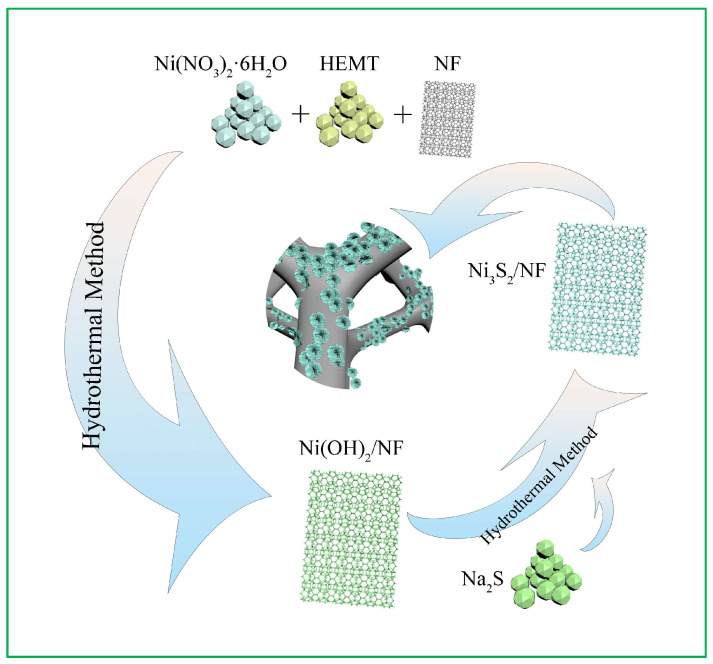
Schematic diagram of the fabrication methodology for the Ni_3_S_2_/NF hybrid.

An overview of the fabrication strategy of hierarchical Ni_3_S_2_ NW on a 3D NF surface to construct a binder-free self-supported nanoarray is provided in Figure 1. This method first outlines the synthesis of Ni(OH)_2_/NF via a hydrothermal method. Next, Na_2_S acts as the sulfur source and S^2−^ ions are released during the hydrolysis of Na_2_S. Ni^2+^ and S^2−^ ions can easily combine to precipitate Ni_3_S_2_ under hydrothermal conditions [29]. Finally, Ni(OH)_2_ and Na_2_S react to form the Ni_3_S_2_ NW via the following reactions:

Na_2_S → 2Na^+^ + S^2−^ (1)

3Ni(OH)_2_ + 2S^2−^ → Ni_3_S_2_ + 6OH^−^ (2)

The nanosized Ni_3_S_2_/NF material was grown vertically in situ on NF. The NF exhibits a typical morphology of a 3D cross-linked porous framework that facilitates ion transport in electrochemical reactions. Its unique structure contains several voids and provides ample active sites for electrochemical reactions [30]. Furthermore, this 3D porous honeycomb-like structure helps avoid the congestion of electrolyte ions and provides a large exposed surface area, ensuring efficient ion diffusion and a sufficient Faradaic redox reaction [14].

**Figure 2:**
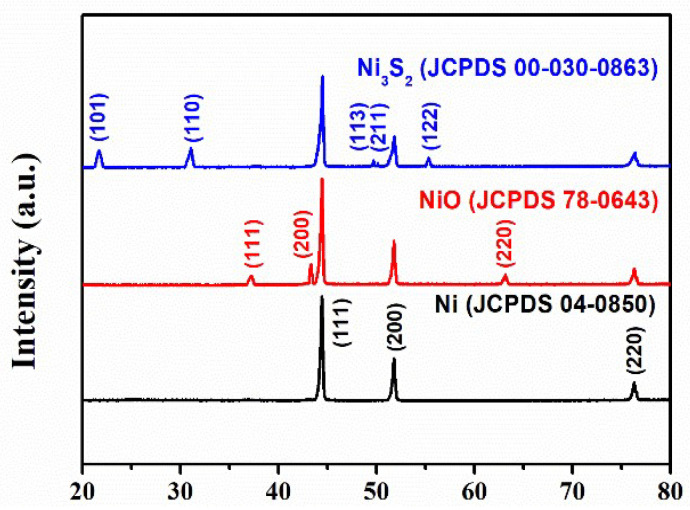
X-ray diffraction (XRD) patterns of nickel foam (NF), NiO/NF, and Ni_3_S_2_/NF.

Figure 2 shows the phase compositions and crystal structures of NF, NiO, and Ni_3_S_2_ based on XRD analyses. The peaks located at 37.27°, 43.35°, and 63.16° were identified as the (111), (200), and (220) planes of NiO (JCPDS 78-0643), respectively. The diffraction peaks at 21.70°, 31.09°, 49.71°, 50.23°, and 55.35° aligned well with the (101), (110), (113), (211), and (122) planes of Ni_3_S_2_ (JCPDS 00-030-0863), respectively. The diffraction peaks located at 44.65°, 52.02°, and 76.51° for NiF (JCPDS 04-0850) were assigned to (111), (200), and (220) crystal planes, respectively. The XRD pattern clearly revealed that all the NiF and NiO/NF were successfully transformed into the hybrid Ni_3_S_2_/NF material.

**Figure 3:**
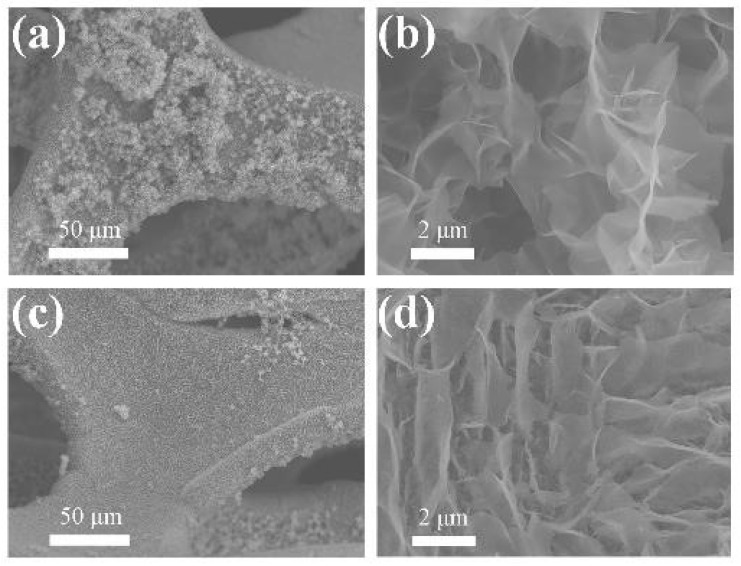
Scanning electron microscopy (SEM) images of (**a**,**b**) NiO/NF and (**c**,**d**) Ni_3_S_2_/NF.

The SEM morphologies of the NiO/NF and Ni_3_S_2_/NF electrodes are shown in Figure 3. After sulfidation, Ni_3_S_2_ NW were uniformly dispersed on the NF and were tightly anchored to the NF networks (Figure 3a,c). Both Figure 3b,d show 3D NW structures. Compared with NiO/NF, Ni_3_S_2_/NF had more tightly bonded NWs and NF; this special structure enables it to withstand more stringent redox reactions in alkaline electrolyte, resulting in enhanced cycling stability.

For further insight into the morphology and microstructure of the as-fabricated Ni_3_S_2_ materials, we performed high-resolution transmission electron microscopy (HRTEM).

**Figure 4:**
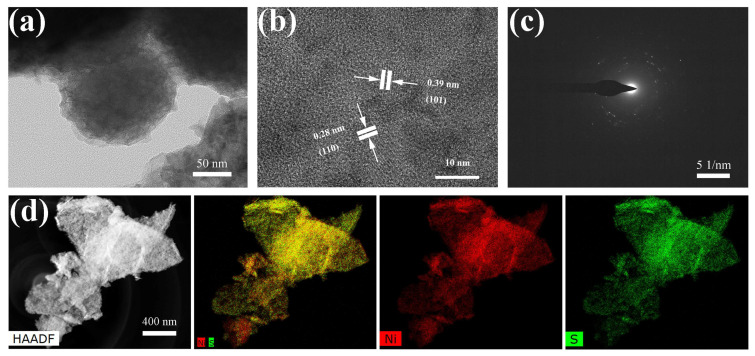
(**a**) Transmission electron microscopy (TEM), (**b**) high-resolution TEM (HRTEM), (**c**) selected-area electron diffraction (SEAD) pattern, (**d**) energy dispersive X-ray spectroscopy (EDS)–high-angle annular dark field images and the corresponding elemental mapping of Ni_3_S_2_.

As shown in Figure 4a, the low-magnification TEM image confirmed the NWs-like structure of Ni_3_S_2_. The HRTEM images shown in Figure 4b present a clear lattice of 0.28 nm, which matched well with the (110) plane of Ni_3_S_2_. The selected-area electron diffraction (SEAD) pattern (Figure 4c) shows several concentric rings, indicating the polycrystalline structure of Ni_3_S_2_ NW [31]. Figure 4d clearly illustrates the uniform distribution of Ni and S elements in the hybrid material.

**Figure 5:**
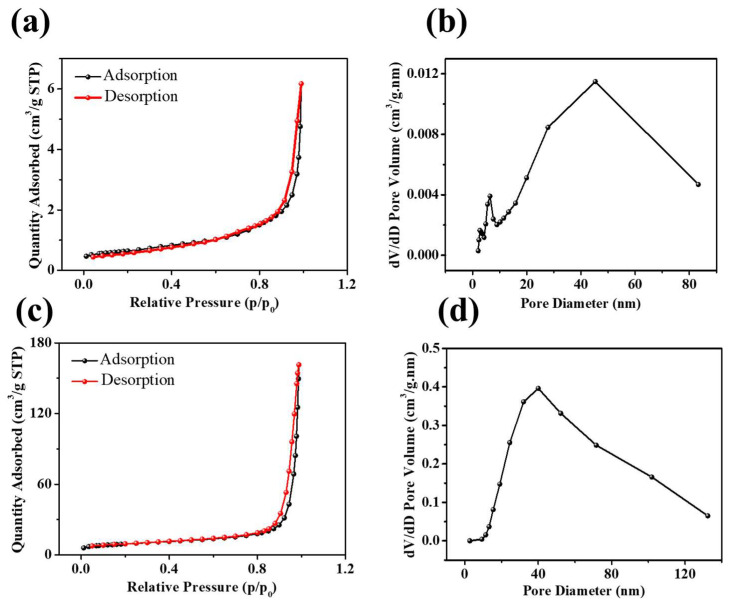
N_2_ adsorption/desorption isotherms of (**a**) NiO and (**c**) Ni_3_S_2_; the pore-size distributions of (**b**) NiO and (**d**) Ni_3_S_2_.

**Figure 6:**
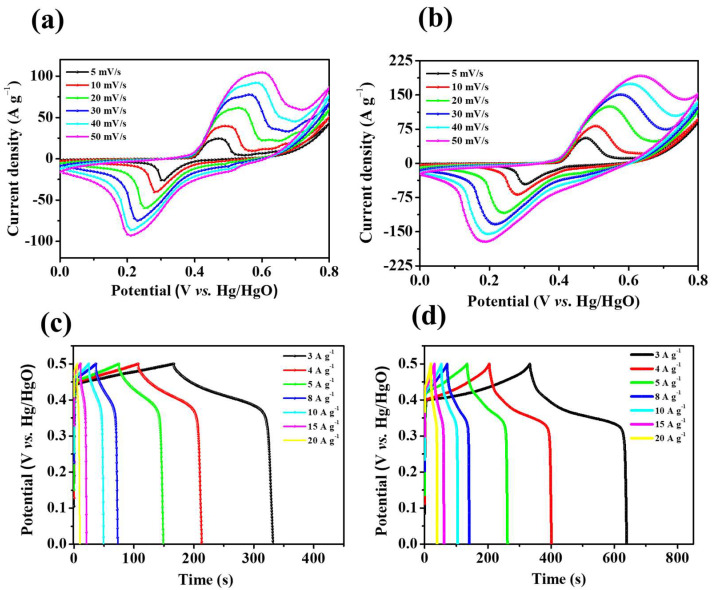
Cyclic voltammogram (CV) curves of (**a**) NiO/NF and (**b**) Ni_3_S_2_/NF, (**c**) GCD curves of NiO/NF and (**d**) Ni_3_S_2_/NF.

The surface area, pore types, and pore-size distributions of NiO and Ni_3_S_2_ were investigated based on nitrogen adsorption/desorption isotherms and the BJH pore-size distribution, as shown in Figure 5. The N_2_ adsorption/desorption isotherms of NiO and Ni_3_S_2_ indicate slit-like pores in the Ni_3_S_2_ mesoporous structure, as shown in Figure 5a,c. The specific BET surface areas of NiO and Ni_3_S_2_ were 2.2 and 32.3 m^2^ g^−1^, respectively, and their average pore sizes were 18 and 37.1 nm, respectively. Figure 3b,d show the BJH (the Barret, Joyner, and Halenda method) pore-size distributions of NiO and Ni_3_S_2_; the three main peaks at 2.7, 6.5, and 45.3 nm for NiO and the local peak at 40.0 nm for Ni_3_S_2_ indicate excess mesoscale pores in both NiO and Ni_3_S_2_. Ashkan et al. [32] studied the Li intercalation/deintercalation in bulk LiCoO_2_ and at the LiCoO_2_ (10$\bar{1}$4) surface using DFT calculations and found that the diffusion barriers between the topmost second and third layers are lower than those in bulk LiCoO_2_. This finding indicated that nanosized LiCoO_2_ with a large surface area/volume ratio is a promising cathode material for fast charging/discharging Li–ion batteries. Wang et al. [33] also suggested that both the electrochemically active surface area and the internal structure contribute to the effective diffusion coefficient. Pseudocapacitors store electric charges via rapid and surface/near-surface controlled non-diffusion limited Faradaic redox reactions. According to Equation (3), a large ion-accessible surface area can generate high capacitance in a certain electrolyte [34].

(3) $$C=\frac{\varepsilon_{r}\varepsilon_{0}}{d}A,$$

where $\varepsilon_{r}$, $\varepsilon_{0}$, *d*, and *A* represent the permittivity of the vacuum (F·m^−1^), the relative dielectric constant of the electrolyte solution, the distance between the electrolyte ions, and the electrode surface (m) and ion-accessible surface area of the electrode material (m^2^), respectively. Considering the results of XRD, SEM, HR-TEM, and BET characterization analyses of NiO and Ni_3_S_2_ electrodes and the simple synthesis process, we believe that the attractive nanostructure can be employed for energy-storage applications. The three electrode-based electrochemical measurements of the Ni_3_S_2_ and NiO electrodes were evaluated using cyclic voltammetry, GCD, and electrochemical impedance spectroscopy (EIS) results obtained for a 1 M KOH aqueous electrolyte solution. Figure 6a,b show a pair of redox peaks in each CV curve, revealing that the typical pseudocapacitance of Ni_3_S_2_ and NiO electrode materials can be attributed to the redox mechanism, and the possible chemical reactions can be described as follows [31,35,36]:

NiO + OH^−^ ←→ NiOOH + e^−^ (4)

Ni_3_S_2_ + 3OH^−^ ←→ Ni_3_S_2_(OH)_3_ + 3e^−^ (5)

**Figure 7:**
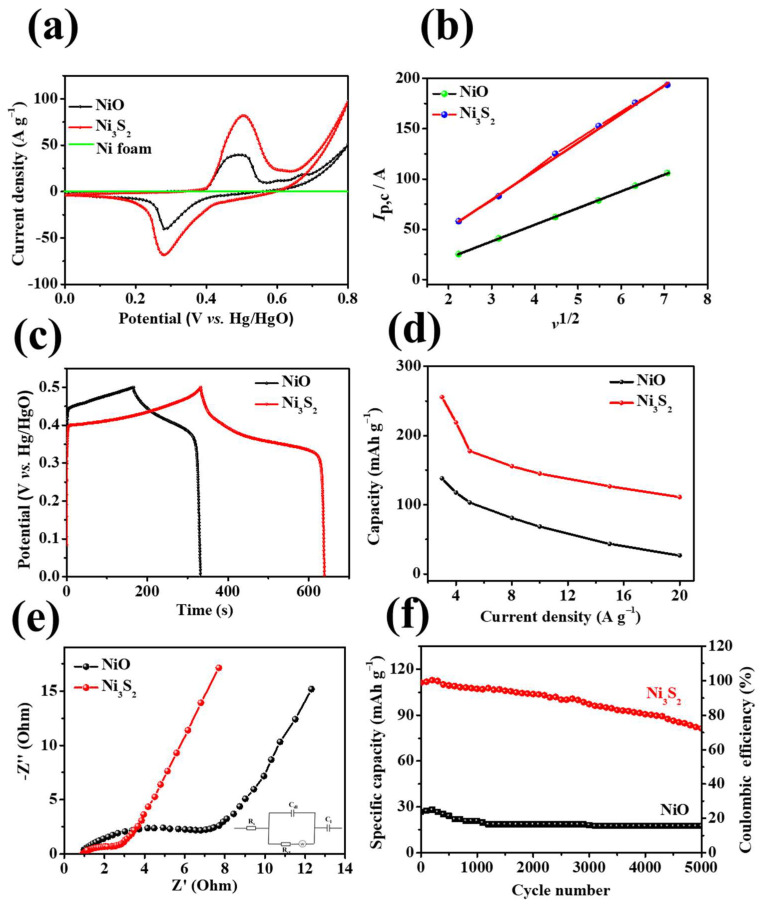
(**a**) CV curves and of NF, NiO/NF, and Ni_3_S_2_/NF at 10 mV s^−1^; (**b**) plot of the current density to the square root of the scan rate, (**c**) GCD curves at 3 A g^−1^, (**d**) specific capacity at various current densities, (**e**) EIS and (**f**) cycling performances of the NiO/NF and Ni_3_S_2_/NF electrodes.

The peak current increases with the scanning rate, indicating that the electrode has high-rate capability [37]. Notably, the peak current value of Ni_3_S_2_ is higher than that of NiO because Ni_3_S_2_ has a dense lamellar structure (as shown in the SEM images in Figure 2), which is conducive to rapid electrolyte–ion transport. Figure 6c,d show the GCD curves of NiO/NF and Ni_3_S_2_/NF at different current densities (3–20 A g^−1^). The GCD curves of both materials exhibit Faraday capacitance characteristics; this finding agrees well with the CV results. Furthermore, the charge/discharge time increased from 332.2 s for NiO/NF to 639.2 s for Ni_3_S_2_/NF at 3 A g^−1^, indicating that the latter had a higher specific capacity. The specific capacity of the Ni_3_S_2_/NF electrode (255.3 mAh g^−1^) was much higher than that of the NiO electrode (138 mAh g^−1^) at a current density of 3 A g^−1^; even at a high current density of 20 A g^−1^, the Ni_3_S_2_/NF hybrid electrode still possessed a high specific capacity of 111.1 mAh g^−1^ (the specific capacity of the NiO/NF electrode was 26.7 mAh g^−1^), indicating the superior electrochemical performance of the Ni_3_S_2_/NF electrode (Figure 7d). During oxidation, the chemical state of S^2−^ does not change, but valence transitions from Ni^0^ to Ni^+^ and further to Ni^3+^ occur sequentially. Hence, we inferred that the reversible Faradaic reactions of Ni_3_S_2_ in KOH aqueous solution can be attributed to the valence transitions of Ni^0^ in Ni_3_S_2_ between Ni^0^ and Ni^3+^. Therefore, according to the results deduced from both CV curves and XPS spectra, the two-step oxidation process and the three-step reverse-reduction process in the reversible Faradaic reaction proceed as shown in Equations (6) and (7), respectively [35]:

(6) $${\mathrm{Ni}}_{3}S_{2}\;\;\overset{{\mathrm{Ni}}^{0}\to {\mathrm{Ni}}^{+}}{\to }\;{\mathrm{Ni}}_{3}S_{2}(\mathrm{OH})\;\overset{{\mathrm{Ni}}^{+}\to {\mathrm{Ni}}^{3+}}{\to }{\mathrm{Ni}}_{3}S_{2}{\left(\mathrm{OH}\right)}_{3}$$

(7) $${\mathrm{Ni}}_{3}S_{2}(\mathrm{OH}{)}_{3}\;\;\overset{{\mathrm{Ni}}^{3+}\to {\mathrm{Ni}}^{2+}}{\to }\;{\mathrm{Ni}}_{3}S_{2}(\mathrm{OH}{)}_{2}\;\overset{{\mathrm{Ni}}^{2+}\to {\mathrm{Ni}}^{+}}{\to }\;{\mathrm{Ni}}_{3}S_{2}(\mathrm{OH})\;\overset{{\mathrm{Ni}}^{+}\to {\mathrm{Ni}}^{0}}{\to }{\mathrm{Ni}}_{3}S_{2}$$

As illustrated in Figure 7a, the CV curve of NF exhibits an approximately linear shape, indicating that the contribution of the NF in the hybrid to the capacity of the material is negligible. Moreover, the CV curve of the Ni_3_S_2_/NF electrode has a considerably more enhanced integral area than that of the NiO/NF electrode, implying that the Ni_3_S_2_/NF electrode has superior specific capacity; this finding is consistent with the GCD curve comparison (Figure 7c). The plots of the current density against the square root of the scan rate for NiO/NF and Ni_3_S_2_/NF are shown in Figure 7b. I_p_ increased linearly with ν^1/2^, confirming that the Faradic capacities of both electrodes were limited by the electrolyte–ion permeation to the active sites. The Randles–Sevcik equation [38,39] was used to compute the diffusion coefficient of the NiO/NF and Ni_3_S_2_/NF electrodes:

(8) $$i_{p}=\left(2.69\times 10^{5}\right)n^{\frac{3}{2}}A{D_{0}}^{\frac{1}{2}}C_{0}^{*}v^{\frac{1}{2}},$$

where *i_p_*, *n*, *A*, *D*_0_, $C_{0}^{*}$, and *ν* are the peak current, the number of electrons transferred, the electrode area, the diffusion coefficient, the reactant concentration, and the scan rate, respectively. The diffusion coefficients (D_NiO/NF_ and D_Ni3S2/NF_) of the NiO/NF and Ni_3_S_2_/NF electrodes were calculated using Equation (9), assuming the same *n*, *A*, and $C_{0}^{*}$ values for both electrodes. The Ni_3_S_2_/NF electrode (D_Ni3S2/NF_) was 2.9 times that of the NiO/NF electrode. This difference is attributed to the compact Ni_3_S_2_ nanostructure, which is conducive to the rapid diffusion of electrolyte ions.

(9) $$\frac{D_{{\mathrm{Ni}}_{3}S_{2}/\mathrm{NF}}}{D_{\mathrm{NiO}}}={\left[\frac{{\left(\frac{i_{p}}{v^{\frac{1}{2}}}\right)}_{{\mathrm{Ni}}_{3}S_{2}/\mathrm{NF}}}{{\left(\frac{i_{p}}{v^{\frac{1}{2}}}\right)}_{\mathrm{NiO}}}\right]}^{2}={\left(\frac{28.46207}{16.62118}\right)}^{2}=2.932$$

The fitted line of the Ni_3_S_2_/NF electrode has a higher slope than that of the NiO/NF electrode, indicating faster ion diffusion kinetics of the Ni_3_S_2_/NF electrode. The outstanding electrochemical performance of the NiO/NF and Ni_3_S_2_/NF samples were further analyzed based on the EIS results shown in Figure 7e. The Nyquist impedance plot (Figure 7e) comprises two parts: a large linear portion in the low frequency range and a semicircular part at high frequencies. In the figure, *Rs* is the equivalent series resistance, which comprises the electrolyte resistance, intrinsic resistance of the active materials, and contact resistance at the interface between the current collector and the active materials. *C_dl_* is the double-layer capacitance, *R_ct_* is the charge transfer resistance, and *Z_W_* is the Warburg impedance. Figure 7e shows the Nyquist plots of NiO/NF and Ni_3_S_2_/NF. *R_ct_* is low in the high frequency region and has a larger slope in the low frequency region for the Ni_3_S_2_/NF electrode, indicating lower charge transfer resistance, lower diffusion resistance, and faster ion transport in the electrolyte in the case of the Ni_3_S_2_/NF electrode [40]. As shown in Figure 7f, the Ni_3_S_2_/NF electrode still retained a specific capacity of 72.17% after 5000 cycles (at a current density of 20 A g^−1^), indicating good cycling stability; this value exceeded that of the NiO/NF electrode (62.38%). The enhanced cycling stability of the Ni_3_S_2_/NF electrode can be attributed to its unique structure, abundant active sites, low resistance, and the synergistic effects of components.

**Figure 8:**
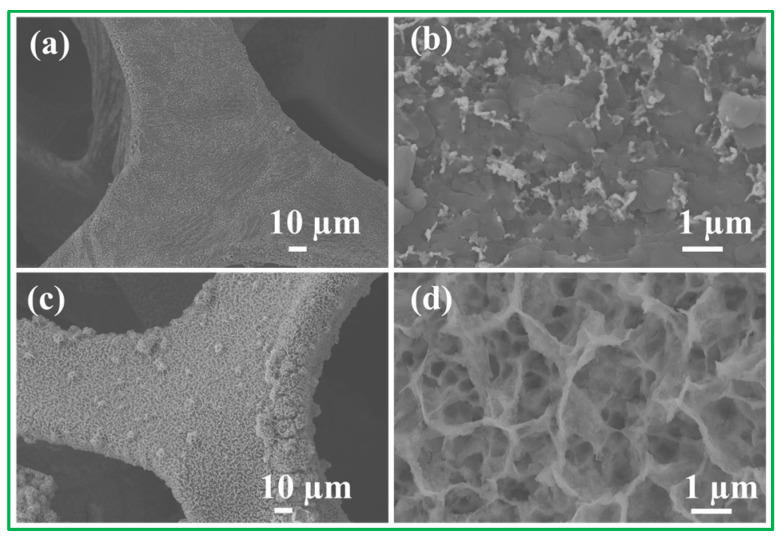
SEM images of (**a**,**b**) NiO/NF and (**c**,**d**) Ni_3_S_2_/NF after 5000 cycles numbers.

Cycling stability is a crucial parameter for evaluating the electrochemical performance of Ni_3_S_2_/NF electrode material. Figure 8a–d show the SEM images of the NiO/NF electrodes and Ni_3_S_2_/NF electrodes after 5000 cycles. The gradual increase in capacitance during the cycles may be attributed to the activation of the Ni_3_S_2_/NF electrode materials through the slow intercalation of the electrolyte into the gaps between the nanowires of Ni_3_S_2_/NF. The low-magnification SEM images in Figure 8a,c indicate that the NiO/NF and Ni_3_S_2_/NF sample can be seen to be different from the previous morphology (Figure 3a,c). The NWs structure of the NiO/NF disappeared completely after 5000 cycles (Figure 8b). The Ni_3_S_2_/NF still maintained its NWs structure after 5000 cycles, exhibiting good cycling stability (Figure 8d). The electrochemical performance of the Ti_3_C_2_T_x_ material was previously reported [41]. Hence, such unexpected cycling stability of the Ni_3_S_2_/NF sample can be attributed to the excellent morphological, structural, and compositional stabilities of the Ni_3_S_2_/NF sample. All the aforementioned findings reveal that the Ni_3_S_2_/NF sample may be a practical candidate for applications in high-performance electrodes for energy storage. This supposition was further established via comparisons with other NiO/NF electrodes and pristine electrode materials. The large surface areas of self-grown Ni_3_S_2_/NF samples are responsible for the enhanced contact between the electrolytes and the Ni_3_S_2_/NF sample. In the Ni_3_S_2_/NF sample, nanowires were self-grown directly on the NF via a hydrothermal method. The Ni_3_S_2_ NWs adhered strongly to the NF. These Ni_3_S_2_ NWs can be used directly as an SC electrode without any binders or conducting agents. Therefore, the hydrothermal route described herein is an environment-friendly approach that is strongly recommended for fabricating other self-supported electrodes.

**Figure 9:**
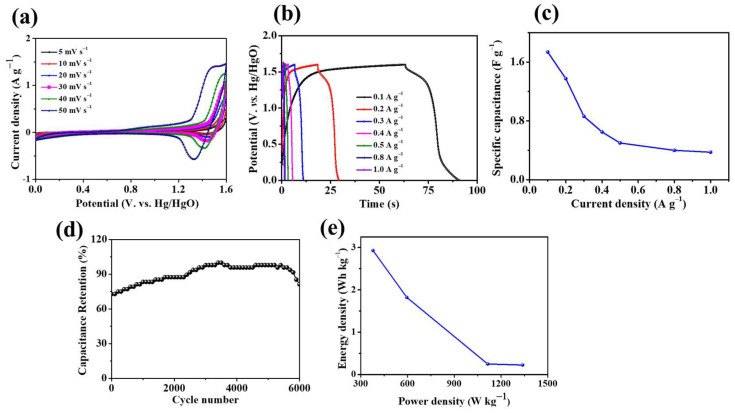
(**a**) CV curves, (**b**) GCD curves, (**c**) specific capacitance, (**d**) cycling performance (at 0.2 A g^−1^), and (**e**) the Ragone plot of the Ni_3_S_2_/NF//Ti_3_C_2_T_x_ ACS device.

To explore the application of Ni_3_S_2_/NF in energy storage applications, we fabricated a Ti_3_C_2_T_x_-based asymmetric coin cell device. In this device, Ni_3_S_2_/NF and Ti_3_C_2_T_x_ served as the positive and negative electrodes, respectively. The electrochemical properties of the Ni_3_S_2_/NF//Ti_3_C_2_T_x_ ASC were tested in a two-electrode system. CV measurements of the Ni_3_S_2_/NF//Ti_3_C_2_T_x_ device were performed at various scan rates (5–50 mV), as shown in Figure 9a. The CV curves revealed features of Faradaic behavior, and the device’s shape was retained during the oxidation and reduction processes with an increasing scan rate. The charge/discharge curves of the device at various current densities are shown in Figure 9b, and they indicate that the device has excellent reversibility and high Coulombic efficiency.

The variation in the specific capacitance of the ASC device with the specific current is illustrated in Figure 9c. The specific capacitance decreased regularly with increasing specific current. As shown in Figure 9d, during the first 3500 cycles, the capacitance retention of the Ni_3_S_2_/NF//Ti_3_C_2_T_x_ increased steadily; this increase may be attributed to the activation process. The capacitance remained stable for the next 2000 cycles and then steadily dropped to 81.25% for the last 500 cycles, thereby demonstrating excellent cycling stability. Interestingly, the SC value of the ASC device initially increased, possibly because the proper wetting of the electrode active materials by KOH electrolyte improved the electrolyte/electrode contact [42]. These results show the superior capability of the Ni_3_S_2_/NF//Ti_3_C_2_T_x_ ASC device with excellent stability and high performance during the long cycle life.

Figure 9e shows that the Ni_3_S_2_/NF//Ti_3_C_2_T_x_ ASC device achieved an energy density of 2.93 Wh kg^−1^ with a power density of 379.16 W kg^−1^. When the power density increased to 1338.83 W kg^−1^, the energy density dropped to 0.22 Wh kg^−1^. The energy density of our device exceeded that of the SCs reported by Xu et al. [43] and Ren et al. [44]; in these previous works, the SCs were based on carbon nanotubes (CNTs)–MnO_2_ fibers (17.26 nWh cm^−1^ with the corresponding power density changing from 61.55 μW cm^−1^) and CNT–OMC fibers (1.26 × 10^−6^ nWh cm^−2^ with the corresponding power density changing from 0.043 mW cm^−2^), respectively. A symmetrical linear SC was assembled by Zhang et al. [45], and it consisted of a CNT–MnO_2_ fiber electrode and a polyvinyl alcohol/H_3_PO_4_ electrolyte and had an energy density of 86 nWh cm^−1^. Table 1 lists the electrochemical performances of various electrode materials for comparison. The excellent electrochemical properties of the Ni_3_S_2_/NF electrode are attributed to its unique structure, with many voids and abundant active sites for electrochemical reaction processes. This 3D porous honeycomb-like structure helps avoid congestion of electrolyte ions and increases the exposed surface area, thereby ensuring efficient ion diffusion and sufficient Faradaic redox reaction.

## 3. Materials and Methods

### 3.1. Preparation of the Nickel Sulphide Electrode

Sodium sulfides were supplied by Sigma Aldrich (St. Louis, MO, USA). NF (110 PPI pore density and a mass density of 320 g m^−2^) were obtained from Artenano Company Limited (Hong Kong). Deionized water (DI) obtained from Millipore was used as a solvent in all experiments. Before use, the NF was prepared for experiment through previously reported routes. Ni foam of area 1 × 1 cm was thoroughly cleaned before the experiment by the following steps: It was degreased by immersion in acetone for 30 min; etched with dilute HCl (3.0 mol L^−1^) for 15 min, and rinsed with DI water before drying. The precursor of Ni(OH)_2_ was described in our previous report [53]. First, 100 mmol of Ni(NO_3_)_2_·6H_2_O and 25 mmol of hexamethylenetetramine were dissolved in 50 mL DI water, and cleaned NFs (2 × 4 cm) were placed in an autoclave at temperatures below 90 °C for 4 h. Subsequently, the as-obtained Ni(OH)_2_/NF substrates were immersed in a 50 mL autoclave with 50 mmol Na_2_S; the autoclave was then heated to 120 °C and maintained at this temperature for 4 h to generate Ni_3_S_2_. After the autoclave was allowed to cool to room temperature (25 °C), the Ni_3_S_2_ was taken out of the autoclave and rinsed separately several times with anhydrous ethanol and distilled water.

### 3.2. Electrode Production

A previously published method [54] was used to clean NF. NiO/NF (1 × 1 cm) and Ni_3_S_2_/NF (1 × 1 cm) were coated with the treated NF. The Ti_3_C_2_T_x_ MXene composite, carbon black, and polytetrafluoroethylene (PTFE) solution (60 wt%) binder were mixed in a mass ratio of 8:1:1 to fabricate the working electrode. The homogeneous slurry was coated on the cleaned NF and dried for 12 h at 60 °C in a vacuum oven.

### 3.3. Fabrication of the ASC Device

The Ni_3_S_2_/NF//Ti_3_C_2_T_x_ MXene ASC device had a mass ratio of 1:6, and its positive and negative electrodes were Ni_3_S_2_/NF and Ti_3_C_2_T_x_ MXene, respectively. From the galvanostatic charge–discharge (GCD) curves, the specific capacitance of ASC (*C_d_*) was calculated as follows [55]:

(10) $$C_{d}=\frac{I\;\Delta t}{M\;\Delta V}$$

where *I* (A), Δ*t* (s), *M* (g), and Δ*V* (V) are the applied discharge current, the discharge time, the total mass of the active material, and the potential window, respectively. The energy density (Wh kg^−1^) and the power density (W kg^−1^) of the ASC device were calculated from the GCD curve using the following equations [52]:

(11) $$E=\frac{\int I\cdot V\left(t\right)dt}{3.6M}$$

*P* = 3600*E*/Δ*t*, (12)

where *E* (Wh kg^−1^), *I* (A), *V*(*t*) (V), *P* (W kg^−1^), *M* (g), and Δ*t* (s) are the energy density, the applied current, the potential window, the power density, the total mass of the active material, and the discharge time of the ASC device, respectively.

### 3.4. Electrochemical Measurements

The X-ray diffraction patterns were collected by an X-ray diffractometer (Rigaku, SmartLab, Tokyo, Japan). Cu Kα X-ray radiation at 40 kV and 40 mA was used to identify the crystal structure and the phase purity of in situ grown Ni_3_S_2_ and NiO. Field-emission scanning electron microscopy (FE-SEM; Merlin Compact, Carl Zeiss NTS GmbH, Oberkochen, Germany), equipped with an instrument for energy dispersive X-ray spectroscopy (EDS), was performed at 15 kV to investigate the surface morphology and the elemental composition of individual nickel halides. Transmission electron microscopy (TEM) and selected-area electron diffraction patterns were operated on an FEI TalosF200x transmission electron microscope at 200 kV to further study the microstructure of the samples. To examine the surface area and pore-size distribution, the Brunauer–Emmett–Teller (BET) and Barrett–Joyner–Halenda (BJH) measurements were conducted using a Micrometrics ASAP2010 analyzer in N_2_ gas under suitable humidity conditions. Electrochemical measurements of Ni_3_S_2_ and NiO electrode materials were conducted using an electrochemical workstation (Ivium vertex, Eindhoven, The Netherlands) based on cyclic voltammogram (CV) and GCD measurements. CV measurements were performed in the range of 0–0.8 V at different scan rates. The GCD scans of the Ni_3_S_2_ and NiO were obtained at various current rates within the potential window of 0–0.6 V in a 1 M KOH aqueous electrolyte solution. Hg/HgO and a platinum foil were used as the reference and counter electrode, respectively. The as-fabricated Ni_3_S_2_//MXene ASC device was assembled and tested in 1 M KOH electrolyte solution comprising a two-electrode system with a separator to avoid short circuiting.

## 4. Conclusions

In summary, a highly hierarchical 3D porous Ni_3_S_2_ nanosheet array was directly grown on NF via a hydrothermal method. This low-cost and simple-synthesis method can be extended to the commercial fabrication of the hybrid material for practical applications. Moreover, the pores among Ni_3_S_2_ NW facilitate electrolyte diffusion and electron transmission. Therefore, the Ni_3_S_2_/NF hybrid meets the requirements of rapid ion diffusion and transportation and shows high specific capacity, excellent rate performance, and good cycling stability; these properties are attributed to the 3D porous structure, the enhanced conductivity, and the facile electrolyte penetration of Ni_3_S_2_ NW. Furthermore, the synthesized Ni_3_S_2_ NW possessed high specific capacitance and excellent stability during electrochemical analysis. Hence, this material may be promising for electrodes in SC applications. The unique architecture of the Ni_3_S_2_ electrode provides excellent electrochemical performance with small charge transfer resistance, which endows the as-prepared Ni_3_S_2_ electrode with high capacitance as well as excellent cycling stability. The above self-growth of the Ni_3_S_2_ electrode makes it appealing for other applications, such as catalysts and sensor batteries. Furthermore, the hydrothermal fabrication method is simple and cost-effective, and the fabricated material is binder-free. This approach can be adopted in the fabrication of other self-supported metal oxide electrodes for SCs or energy storage applications.

## Figures and Tables

**Table 1:** Comparison of the electrochemical performances of the various electrode materials.

Electrode Material	Electrolyte	Capacitance (Cs/Scan Rate)	Refs.
NiMoO_4_@NiWO_4_	3 M KOH	1290 F g^−1^/2 A g^−1^	[46]
Co_9_S_8_@Ni(OH)_2_	2 M KOH	1620 F g^−1^/0.5 A g^−1^	[47]
NiS@NiSe_2_	2 M KOH	1412 F g^−1^/0.5 A g^−1^	[48]
Ni_3_S_2_@Co_9_S_8_	2 M KOH	925 F g^−1^/0.5 A g^−1^	[48]
Ni_3_S_4_@rGO	2 M KOH	1830 F g^−1^/2 A g^−1^	[49]
Co-Ni_3_S_2_	2 M KOH	1075.5 F g^−1^/1 A g^−1^	[50]
Ni_3_S_2_@NF	1 M KOH	736.64 F g^−1^/0.8 A g^−1^	[51]
NiS_2_/Ti_3_C_2_T_x_	1 M KOH	72.0 mAh g^−1^/1 A g^−1^	[52]
Ni_3_S_2_@Ni	6 M KOH	945.71 F g^−1^/17.15 A g^−1^	[35]
Ni_3_S_2_/NF	1 M KOH	1839.6 F g^−1^ (Corresponding specific capacity: 255.5 mAh g^−1^/3 A g^−1^)	This work
